# Alkaloids and Styryl lactones from *Goniothalamus ridleyi* King and Their *α*-Glucosidase Inhibitory Activity

**DOI:** 10.3390/molecules28031158

**Published:** 2023-01-24

**Authors:** Isaraporn Polbuppha, Passakorn Teerapongpisan, Piyaporn Phukhatmuen, Virayu Suthiphasilp, Tharakorn Maneerat, Rawiwan Charoensup, Raymond J. Andersen, Surat Laphookhieo

**Affiliations:** 1 Center of Chemical Innovation for Sustainability (CIS) and School of Science, Mae Fah Luang University, Chiang Rai 57100, Thailand; 2 Department of Industrial Technology and Innovation Management, Faculty of Science and Technology, Pathumwan Institute of Technology, Bangkok 10330, Thailand; 3 Medicinal Plant Innovation Center, Mae Fah Luang University, Chiang Rai 57100, Thailand; 4 School of Integrative Medicine, Mae Fah Luang University, Chiang Rai 57100, Thailand; 5 Department of Chemistry and Department of Earth, Ocean & Atmospheric Sciences, University of British Columbia, 2036, Main Mall, Vancouver, BC V6T 1Z1, Canada

**Keywords:** *Goniothalamus ridleyi*, dimeric aristolactam, styryl lactone, *α*-glucosidase inhibitory activity

## Abstract

Gonioridleylactam (**1**), a new compound, is a unique dimeric aristolactam isolated from the EtOAc extract of the twigs of *Goniothalamus ridleyi* King. The structure of gonioridleylactam (**1**) consists of two different aristolactams linked together with two methylenedioxy bridges at C–3/C–3′ and C–4/C–4′, generating a ten-membered ring of [1,3,6,8]tetraoxecine. A new natural product, gonioridleyindole (3-hydroxymethyl-1-methyl-1*H*-benz[*f*]indole-4,9-dione, **2**), together with eight known compounds (**3–10**) were also isolated from this plant. Their structures were extensively characterized by spectroscopic methods and comparisons were made with the literature. Compounds **1–4**, **7**, and **9** were evaluated for their *α*-glucosidase inhibitory activity. Of these, 3,5-demethoxypiperolide (**7**) displayed the highest *α*-glucosidase inhibitory activity, with an IC_50_ value of 1.25 µM.

## 1. Introduction

*Goniothalamus ridleyi* King belongs to the Annonaceae family, widely distributed throughout Thailand (southern part), Peninsular Malaysia, and Sumatra. Plants in the *Goniothalamus* genus produce diverse bioactive compounds, including styryl lactones, acetogenins, flavonoids, and alkaloids [1,2,3,4]. Many of these compounds have been reported to exhibit cytotoxic, antibacterial, larvicidal, and antitubercular activities [3,4,5,6,7]. For example, two styryl lactones, (−)-5-acetoxygoniothalamin and (*Z*)-6-styryl-5,6-dihydro-2-pyranone, displayed cytotoxicity against a colon cancer cell line (HCT116), with IC_50_ values of 8.6 and 22.2 μM, respectively [4]. (+)-3-Acetylaltholactone and (−)-nordicentrine showed antiplasmodial activity against the parasite *Plasmodium falciparum*, with IC_50_ values of 2.6 and 0.3 μg/mL, respectively [8].

From a previous phytochemical investigation of *G. ridleyi*, five simple styryl lactones [9,10] and 5-hydroxy-6-[(*E*)-2-phenylethenyl]-5,6-dihydro-2*H*-pyran-2-one were isolated and identified. A preliminary bioassay screen conducted by our group revealed that the EtOAc extract of *G. ridleyi* twigs inhibited *α*-glucosidase activity (97% at 250 μg/mL), prompting us to isolate the *α*-glucosidase inhibitory compounds from this plant. This paper describes the isolation, structure elucidation, and *α*-glucosidase inhibitory activity of compounds from *G. ridleyi*.

## 2. Results and Discussion

**Figure 1:**
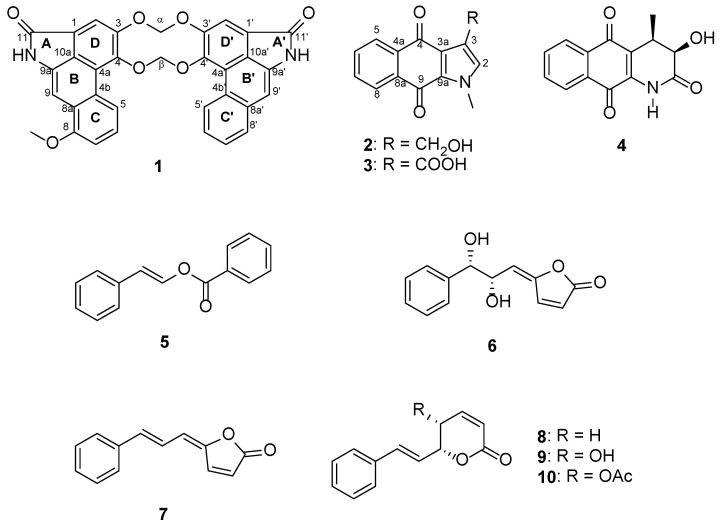
Compounds isolated from the twig extract of *G. ridleyi*.

The EtOAc extract of the twig of *G. ridleyi* was separated and purified by various chromatographic techniques to afford a new dimeric aristrolactam (**1**), a new natural indole alkaloid (**2**), and eight known compounds (**3–10**) (Figure 1). The known compounds were identified as goniochelienic acid B (**3**) [3], griffithazanone A (**4**) [11], ethenol (**5**) [12], goniobutenolide B (**6**) [13], (5-(3-phenyl-2-propenylidene)-2(5H)-furanone) (**7**) [14], (−)-goniothalamin (**8**) [15], (−)-5-hydroxygoniothalamin (**9**) [16], and (−)-5-acetyl goniothalamin (**10**) [17] by extensive NMR spectroscopic data and comparisons made with spectroscopic data reported in the literature.

### 2.1. Structural Elucidation

**Figure 2:**
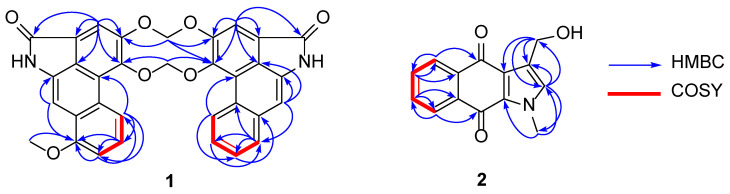
COSY (^1^H—^1^H) and selected HMBC (^1^H→^13^C) correlations of **1** and **2**.

**Figure 3:**
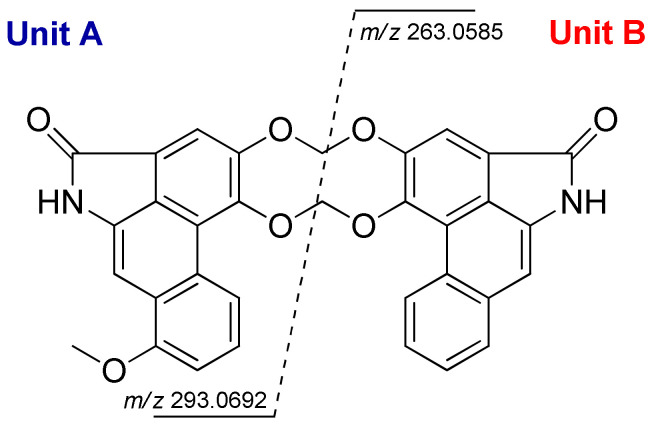
Mass spectral fragmentation of **1**.

Gonioridleylactam (**1**) was isolated as a yellowish solid. The HRESITOFMS spectrum of **1** displayed a sodium adduct ion at *m*/*z* 556.1271, corresponding to the molecular formula of C_33_H_20_N_2_O_7_. The UV spectrum showed maxima absorption bands at *λ*_max_ 250, 277, and 322 nm, while the IR spectrum displayed the presence of NH (3394 cm^−1^) and amide carbonyl (1688 cm^−1^) functionalities. Following an intensive analysis of NMR spectroscopic data, the structure of **1** was determined as a dimeric aristolactam linked together with two different aristolactams (aristolactam units A and B). The ^13^C NMR data, in combination with DEPT and HMQC, displayed 33 carbon resonances, including 1 methyl (*δ*_C_ 55.3), 2 methylenes (*δ*_C_ 103.3 (×2)), 11 methines (*δ*_C_ 128.7, 127.6, 126.8, 125.6, 125.2, 119.2, 108.0, 105.3, 105.2, 104.2, and 98.1), and 17 non-protonated carbons (*δ*_C_ 167.9 (×2), 155.9, 149.3, 149.2, 147.5, 147.3, 135.5, 134.6, 134.7, 125.5 (×2), 124.8, 124.7, 119.8 (×2), 111.8 (×2), and 104.2). Aristolactam unit A displayed the ^1^H and ^13^C NMR resonances as follows: a singlet N*H* proton [*δ*_H_ 9.74 (br s, NH′)], a set of ABC aromatic protons [*δ*_H_ 8.25 (d, *J* = 8.2 Hz, H-5)/*δ*_C_ 119.2, 7.52 (t, *J* = 8.2 Hz, H-6)/*δ*_C_ 125.6, and 7.20 (d, *J* = 8.2 Hz, H-7)/*δ*_C_ 108.0], two singlet aromatic protons [*δ*_H_ 7.58 (s, H-2)/*δ*_C_ 105.0, and 7.54 (1H, s, H-9)/*δ*_C_ 98.1], and one methoxy group [*δ*_H_ 4.05 (s, 8-OMe)/*δ*_C_ 55.3]. The methoxy group was placed at C-8 due to the HMBC cross peaks between H-6, H-7, MeO-8, and H-9 with C-8 (*δ*_C_ 155.9) (Figure 2 and Table 1). In the case of aristolactam unit B, the ^1^H and ^13^C NMR data were similar to those of aristolactam unit A. The main difference between aristolactam units A and B is that the OMe-8 resonance of aristolactam unit B was not observed. Aristolactam unit B displayed four aromatic protons of 1,2-disubstituted benzene at *δ*_H_ 8.63 (1H, dd, *J* = 7.8, 1.5 Hz, H-5′)/*δ*_C_ 126.8, 7.58 (1H, td, *J* = 7.8, 1.5 Hz, H-6′)/*δ*_C_ 125.2, 7.61 (1H, td, *J* = 7.8, 1.5 Hz, H-7′)/*δ*_C_ 127.6, and 7.92 (1H, dd, *J* = 7.8, 1.5 Hz, H-8′)/*δ*_C_ 128.7. These were supported by HMBC correlations from H-5′ (*δ*_H_ 8.63) to C-4a′ (*δ*_C_ 111.8), C-7′ (*δ*_C_ 127.6), and C-8a′ (*δ*_C_ 134.7), from H-6′ (*δ*_H_ 7.58) to C-4b′ (*δ*_C_ 124.8), C-7′ (*δ*_C_ 127.6), from H-7′ (*δ*_H_ 7.61) to C-8a′ (*δ*_C_ 134.7), C-5′ (*δ*_C_ 126.8), and from H-8′ (*δ*_H_ 7.92) to C-4b′ (*δ*_C_ 124.8), C-6′ (*δ*_C_ 125.2), and C-9′ (*δ*_C_ 104.2) (Figure 2). Aristolactam units A and B linked together with two methylenedioxy bridges at C–3/C–3′ [6.49 (2H, s, H-α/103.3)] and C–4/C–4′ [6.50 (2H, s, H-β/103.3)], generating a ten-membered ring of [1,3,6,8]tetraoxecine. The observed HMBC cross peaks of H-α (*δ*_H_ 6.49) to C-3 (*δ*_C_ 147.3) and C-3′ (*δ*_C_ 147.5) and H-β (*δ*_H_ 6.50) to C-4 (*δ*_C_ 149.2) and C-4′ (*δ*_C_ 149.3) (Figure 2) supported these assignments. In addition, the HRESITOFMS ions of aristolactam units A and B at *m*/*z* 293.0692 and 263.0585 (Figure 3), respectively (Figures S10 and S11, Supplementary Materials), also supported the linkage of aristolactam units A and B. The full assignment of NMR data and HMBC correlations is shown in Figure 2 and Table 1, respectively. The structure of **1** was the first example of a ten-membered ring of [1,3,6,8]tetraoxecine dimeric aristolactam found in Annonaceae.

The HRESITOFMS spectrum of compound **2**, a new natural product, displayed an [M + Na]^+^ ion at *m*/*z* 264.0635 (Figure S18, Supplementary Materials), corresponding to the molecular formula of C_14_H_11_NO_3_ (Tgt. for C_14_H_11_NO_3_, 241.0742). The UV spectrum showed maxima absorption bands at *λ_max_* 250, 277, and 322 nm suggesting the benzo[*f*]indole-4,9-dione framework [3], while the IR spectrum revealed absorption bands for carbonyl (1709 cm^−1^) and hydroxy (3404 cm^−1^) functionalities. The ^1^H and ^13^C NMR spectroscopic data of **2** (Table 2) showed resonances for four aromatic protons of 1,2-disubstituted benzene [*δ*_H_ 7.70 (m, H-5 and H-8)/*δ*_C_ 126.8 (C-5), 126.7 (C-8) and 8.16 (m, H-6 and H-7)/*δ*_C_ 133.6 (C-6) and 133.3 (C-7)], an olefinic proton [*δ*_H_ 6.85 (s, H-2)/*δ*_C_ 129.3], a hydroxymethylene proton [*δ*_H_ 4.72 (s, H_2_-1′/*δ*_C_ 57.1)], and an *N*-methyl group [*δ*_H_ 4.07 (s, *N*-CH_3_)/*δ*_C_ 36.8]. The structure of **2** was further supported by the following key HMBC correlations (Figure 2): *δ*_H_ 6.85 (H-2) with C-3 (*δ*_C_ 126.2), C-1′ (*δ*_C_ 36.8), and *N*-CH_3_ (*δ*_C_ 57.1); *δ*_H_ 4.72 (H_2_-1′) with C-2 (*δ*_C_ 129.3), C-3 (*δ*_C_ 126.2), and C-3a (*δ*_C_ 162.2); *δ*_H_ 4.15 (*N*-CH_3_) with C-2 (*δ*_C_ 129.3) and C-9a (*δ*_C_ 131.8). Thus, compound **2** was named as gonioridleyindole (3-hydroxymethyl-1-methyl-1*H*-benzo[*f*]indole-4,9-dione). Many 3-hydroxymethylindolequinone derivatives, including compound **2**, had previously been synthesized and functionalized to other indolequinones [18]. However, compound **2** was first isolated from nature.

### 2.2. α-Glucosidase Inhibitory Activity

α-Glucosidase is a carbohydrate hydrolyzing enzyme that maintains postprandial blood glucose and insulin levels [19]. The inhibition of this enzyme can delay intestinal carbohydrate digestion to control hyperglycemia in diabetes mellitus [20]. Exploration for new α-glucosidase inhibitors and other antidiabetic drugs from natural sources has increased in recent years [21]. There are reports of the presence of α-glucosidase inhibitors such as flavonoids (quercetin) [22], terpenoids (wallitaxanes) [23], and alkaloids (5-hydroxynoracronycin) [19]. In this study, compounds isolated with a sufficient amount, **1–4**, **7**, and **9**, were evaluated for their *α*-glucosidase inhibitory activity. Of these, 3,5-demethoxypiperolid (**7**) showed the highest *α*-glucosidase inhibitory activity with an IC_50_ value of 1.25 µM, which is better than that of the standard control (acarbose). Other tested compounds were weak or inactive (Table 3). The observed α-glucosidase inhibitory activity of 3,5-demethoxypiperolid (**7**) indicates that this compound may have the potential as a lead compound for the further development of anti-diabetes agents.

## 3. Conclusions

Phytochemical investigation of the EtOAc extract of the twigs of *Goniothalamus ridleyi* King resulted in the discovery of a unique dimeric aristolactam and nine other compounds. The dimeric aristolactam contained two different aristolactam units linked together with two methylenedioxy bridges forming a [1,3,6,8]tetraoxecine ten-membered ring. The discovery of styryl lactones and alkaloids in this study as the major compounds was in good agreement with previous reports. In addition, the result of preliminary *α*-glucosidase inhibitory assay suggested that (5-(3-phenyl-2-propenylidene)-2(5H)-furanone) may have potential as the lead compound for the development of *α*-glucosidase inhibitory agent.

## 4. Materials and Methods

### 4.1. General Experimental Procedures

Melting points were measured with a Büchi B-540 melting point apparatus (Flawil, Sankt Gallen, Switzerland). UV–vis spectra were recorded with a Varian Cary 5000 UV–vis–NIR spectrophotometer (Agilent Technologies, Santa Clara, CA, USA). The IR spectra were recorded using a Perkin-Elmer FTS FT-IR spectrophotometer (Waltham, MA, USA). The NMR spectra were measured using 400, 500, or 600 MHz Bruker spectrometers (Billerica, MA, USA). HRESITOFMS and LRESIMS spectra were carried out on a Bruker-Hewlett-Packard 1100 Esquire-LC system mass spectrometer (Billerica, MA, USA) and Waters 2695 HPLC (Milford, MA, USA), Waters ZQ equipped with ESCI ion source mass spectrometer, respectively. All quick column chromatography (QCC) and column chromatography (CC) were carried out on silica gel 60 (5–40 μm, SiliCycle Inc., Québec, QC G1P 4S6, Canada) and silica gel 100 (63–200 μm, SiliCycle Inc.), respectively. Sephadex LH-20, when indicated, was also used for CC. Precoated thin-layer chromatography (TLC) plates of silica gel 60 F_254_ were used for analytical purposes.

### 4.2. Plant Material

The twigs of *G. ridleyi* [24] were collected in April 2021 from Narathiwat Province, Thailand. This plant was identified by Mr. Abdulromae Baka (Independent Research Group on Plant Diversity in Thailand, Sichon, Nakhon Si Thammarat, 80120, Thailand). A voucher specimen (MFU-NPR0206) was deposited at the Natural Products Research Laboratory, School of Science, Mae Fah Luang University. Plant materials were dried and stored at room temperature.

### 4.3. Extraction and Isolation

Air-dried twigs of *G. ridleyi* (1.5 kg) were extracted with EtOAc (3 × 20 L) at room temperature and concentrated under reduced pressure to give an EtOAc extract (58.5 g). The twig extract was subjected to quick column chromatography (QCC) over silica gel (100% hexanes to 100% acetone) to give seven fractions (1A–1G). Fraction 1C (5.3 g) was subjected to CC over Sephadex LH-20 CC (100% MeOH) to give four subfractions (2A–2D). Subfraction 2B (3.6 g) was further purified by silica gel CC (1:9 *v*/*v,* acetone–hexanes) to give compound **5** (5.8 mg) and four subfractions (3A–3D). Compound **7** (3.1 mg) was obtained from subfraction 3B (97.2 mg) by silica gel CC (100% CH_2_Cl_2_), while compound **6** (1.0 mg) was isolated from subfraction 3C (68.2 mg) by silica gel CC (1:19 *v*/*v*, acetone-hexanes). Subfraction 1D (2.6 g) was further separated by CC over Sephadex LH-20 (100% MeOH) to afford five subfractions (4A–4E). Purification of subfraction 4D (53.5 mg) by silica gel CC (100% CH_2_Cl_2_) yielded compound **8** (1.5 mg). Fraction 1E (23.5 g) was subjected to QCC over silica gel (100% hexanes to 100% EtOAc) to give four subfractions (5A–5D). Upon standing at room temperature, the white solid was precipitate from subfraction 5B (778.9 mg), which was washed by MeOH to give compound **10** (5.7 mg). Subfraction 5C (13.0 g) was separated by CC over Sephadex LH-20 (100% MeOH) to give four subfractions (6A–6D). Subfraction 6B (1.0 g) was further separated by CC over Sephadex LH-20 CC (100% MeOH) to afford compound **3** (1.9 mg) and two subfractions (7A–7B). Subfraction 7A (887.4 mg) was purified by silica gel CC (1:24 *v*/*v*, EtOAc–CH_2_Cl_2_) to give compound **9** (1.2 mg). Compounds **2** (3.5 mg) and **4** (2.2 mg) were obtained from subfraction 7B (18.6 mg) by silica gel CC (1:24 *v*/*v*, EtOAc–CH_2_Cl_2_). Purification of subfraction 6D (4.3 mg) by silica gel CC (1:24 *v*/*v*, EtOAc-CH_2_Cl_2_) gave compound **1** (2.9 mg) (%yield = 0.019%).

#### 4.3.1. Gonioridleylactam (**1**)

Yellow solid; mp 296–298 °C; UV (MeOH) *λ*_max_ (log *ε*) 259 (4.7), 276 (4.5), 287 (4.5), 328 (4.1), and 390 (4.0) nm; IR (neat) *v*_max_ 3394, 2923, 1688, 1376, 1261, 1093, 1261, 1041, and 801 cm^−1^; ^1^H NMR (600 MHz, acetone-*d*_6_) and ^13^C NMR (150 MHz, acetone-*d*_6_), see Table 1; HRESITOFMS *m/z* 579.1162 [M + Na]^+^ (C_33_H_20_N_2_O_7_, 556.1271, Tgt. Mass, 556.1271), see Figure S8, Supplementary Materials.

#### 4.3.2. Gonioridleyindole (3-Hydroxymethyl-1-methyl-1H-benz[f]indole-4,9-dione, **2**)

Yellow solid; mp 218–220 °C; UV (MeOH) *λ*_max_ (log *ε*) 250 (4.5), 277 (4.2), 332 (3.8), and 399 (2.4) nm; IR (neat) *v*_max_ 3404, 2984, 1709, 1377, 1300, 1191, 1104, 1035, 969, and 693 cm^−1^; ^1^H NMR (400 MHz, CDCl_3_) and ^13^C NMR (100 MHz, CDCl_3_), see Table 2; HRESITOFMS *m/z* 264.0635 [M + Na]^+^ (C_14_H_11_NO_3_, 241.0742, Tgt. Mass, C_14_H_11_NO_3_, 241.0742), see Figure S18, Supplementary Materials.

### 4.4. α-Glucosidase Inhibitory Activity

The previously reported approach was used to perform a colorimetric *α*-glucosidase assay [25]. Briefly, the tested samples (50 µL) were combined with 50 µL of the α-glucosidase enzyme solution (0.05 U/mL) and preincubated at 37 °C for 5 min. The substrate (50 µL, *c* 1 mM), *p*-nitrophenyl *α*-*D*-glucoside, was added and incubated at 37 °C for 30 min. Then, 50 µL of Na_2_CO_3_ (0.3 M) was added. The absorption of the mixture was measured at 405 nm. Acarbose was used as a positive control (185.7 µM).

## Figures and Tables

**Table 1:** ^1^H (600 MHz) and ^13^C (150 MHz) NMR spectroscopic data of **1** in acetone-*d_6_*.

Position	*δ*_C_	*δ*_H_ [mult, *J* in Hz]	HMBC (^1^H→^13^C)
1	119.8		
2	105.0	7.58 (s)	1, 3, 4, 4a, 11
3	147.3		
4	149.2		
4a	111.8		
4b	124.7		
5	119.2	8.25 (d, 8.2)	4b, 6, 7, 8
6	125.6	7.52 (t, 8.2)	5, 7, 8
7	108.0	7.20 (d, 8.2)	5, 6, 8
8	155.9		
8a	104.2		
9	98.1	7.54 (s)	8, 9a,10a
9a	134.6		
10a	125.5		
11	167.9		
1′	119.8		
2′	105.3	7.57 (s)	1′, 3′, 4′, 10a′, 11′
3′	147.5		
4′	149.3		
4a′	111.8		
4b′	124.8		
5′	126.8	8.63 (dd, 7.8, 1.5)	4a′, 7′, 8a′
6′	125.2	7.58 (td, 7.8, 1.5)	8′
7′	127.6	7.61 (td, 7.8, 1.5)	5′, 8a′
8′	128.7	7.92 (dd, 7.8, 1.5)	4b′, 6′, 9′
8a′	134.7		
9′	104.2	7.16 (s)	4a′, 8′, 9a′, 10a′
9a′	135.5		
10a′	125.5		
11′	167.9		
α	103.3	6.49 (s)	3, 3′
β	103.3	6.50 (s)	4, 4′
*N*-H		9.76 (s)	
*N*-H′		9.74 (s)	
8-OMe	55.3	4.05 (s)	8′

**Table 2:** ^1^H (400 MHz) and ^13^C (MHz) NMR spectroscopic data of **2** in CDCl_3_.

Position	*δ*_C_	*δ*_H_ [mult, *J* in Hz]	HMBC (^1^H→^13^C)
2	129.3	6.85 (s)	3, 3a, 9a, 1′, *N*-CH_3_
3	126.2		
3a	162.2		
4	183.0		
4a	133.9		
5	126.8	7.70 (m)	4, 6
6	133.6	8.16 (m)	4a, 5
7	133.3	8.16 (m)	8, 8a
8	126.7	7.70 (m)	7, 9
8a	133.8		
9	176.4		
9a	131.8		
1′	36.8	4.72 (s)	2, 3, 3a
*N*-CH_3_	57.1	4.07 (s)	2, 9a

**Table 3:** *α*-Glucosidase inhibitory activity of some isolated compounds from *G. ridleyi*.

Compounds	%Inhibition at 250 µg/mL	IC_50_, µM
**1**	99.6	138.9 ± 0.9
**2**	99.3	inactive
**3**	99.5	inactive
**4**	98.0	inactive
**7**	98.6	1.25 ± 0.4
**9**	99.8	inactive
Acarbose	88.1	185.7 ± 0.3

## Data Availability

Not applicable.

## Supplementary Materials

The following supporting information can be downloaded at: https://www.mdpi.com/article/10.3390/molecules28031158/s1, Figure S1: ^1^H NMR (600 MHz, acetone-*d*_6_) of gonioridleyilactam (**1**); Figure S2: ^13^C NMR (150 MHz, acetone-*d*_6_) of gonioridleyilactam (**1**); Figure S3: HSQC of gonioridleyilactam (**1**); Figure S4: HMBC of gonioridleyilactam (**1**); Figure S5: COSY of gonioridleyilactam (**1**); Figure S6: NOESY of gonioridleyilactam (**1**); Figure S7: LRESIMS (low-resolution electrospray ionization mass spectrometry) spectrum of gonioridleyilactam (**1**); Figure S8: HRESIMS spectrum of gonioridleyilactam (**1**); Figure S9: HRESIMS spectrum of Unit A (aristolactam II) in **1**; Figure S10: HRESIMS spectrum of Unit B (aristolactam I) in **1**; Figure S11: IR spectrum of gonioridleyilactam (**1**); Figure S12: ^1^H NMR (400 MHz, CDCl_3_) of 3-hydroxymethyl-1-methyl-1*H*-benz[*f*]indole-4,9-dione (**2**); Figure S13: ^13^C NMR (100 MHz, CDCl_3_) of 3-hydroxymethyl-1-methyl-1*H*-benz[*f*]indole-4,9-dione (**2**); Figure S14: HSQC of 3-hydroxymethyl-1-methyl-1*H*-benz[*f*]indole-4,9-dione (**2**); Figure S15: HMBC of 3-hydroxymethyl-1-methyl-1*H*-benz[*f*]indole-4,9-dione (**2**); Figure S16: COSY of 3-hydroxymethyl-1-methyl-1*H*-benz[*f*]indole-4,9-dione (**2**); Figure S17: NOESY of 3-hydroxymethyl-1-methyl-1*H*-benz[*f*]indole-4,9-dione (**2**); Figure S18: HRESIMS spectrum of 3-hydroxymethyl-1-methyl-1*H*-benz[*f*]indole-4,9-dione (**2**); Figure S19: IR spectrum of 3-hydroxymethyl-1-methyl-1*H*-benz[*f*]indole-4,9-dione (**2**); Figure S20: The isolation and purification of isolated compounds **1–10** from the twig extract of *G. ridleyi.*

## Sample Availability

Samples of the compounds are not available from the authors.
