# Supplementary material for: Alkaloids and Styryl lactones from Goniothalamus ridleyi King and Their α-Glucosidase Inhibitory Activity
Source: Molecules. 2023 Jan 24;28(3):1158. doi: 10.3390/molecules28031158 (PMC9918889; doi:10.3390/molecules28031158)
Supplement: Supplementary file 1 [file molecules-28-01158-s001.zip › molecules-2147998-supplementary.pdf]

SUPPLEMENTARY MATERIALS

Alkaloids and styryllactones from *Goniothalamus ridleyi* King and their *α*-glucosidase inhibitory activity

Isaraporn Polbuppha [1], Passakorn Teerapongpisan [1], Piyaporn Phukhatmuen [1], Virayu Suthiphasilp [2], Tharakorn Maneerat [1,3], Rawiwan Charoensup [3,4], Raymond J. Andersen [5,]* and Surat Laphookhieo [1,3,]*

[1]Center of Chemical Innovation for Sustainability (CIS) and School of Science, Mae Fah Luang University, Chiang Rai 57100, Thailand

[2]Department of Industrial Technology and Innovation Management, Faculty of Science and Technology, Pathumwan Institute of Technology, Bangkok 10330, Thailand

[3]Medicinal Plant Innovation Center of Mae Fah Luang University, Chiang Rai 57100, Thailand

[4]School of Integrative Medicine, Mae Fah Luang University, Chiang Rai 57100, Thailand

[5]Department of Chemistry and Department of Earth, Ocean & Atmospheric Sciences, University of British Columbia, 2036, Main Mall, Vancouver, BC, V6T 1Z1, Canada

Abstract: Gonioridleylactam (**1**), an undescribed compound, is a unique dimeric aristolactam isolated from the EtOAc extract of the twigs of *Goniothalamus ridleyi* King. The structure of gonioridleylactam (**1**) consists of two different aristolactams jointed together *via* two methylenedioxy bridges at C–3/C–3′ and C–4/C–4′ generating a ten-membered ring of [1,3,6,8]tetraoxecine. An undescribed natural product, gonioridleyindole (3-hydroxymethyl-1-methyl-1*H*-benz[*f*]indole-4,9-dione, **2**), together with eight known compounds (**3-10**) were also isolated from this plant. Their structures were extensively characterized by spectroscopic methods and comparisons made from the literature. Compounds **1-4**, **7**, and **9** were evaluated for their *α*-glucosidase inhibitory activity. Of these, 3,5-demethoxypiperolide (**7**) displayed the highest *α*-glucosidase inhibitory activity with an IC_{50} value of 1.25 *μ*M.

***** Correspondence:

Assoc. Prof. Dr. Surat Laphookhieo (S.L.); e-mail: surat.lap@mfu.ac.th

Prof. Dr. Raymond J. Andersen (R.J.A.); e-mail address: raymond.andersen@ubc.ca

Contents **Page**

Figure S1 ^{1}H NMR (600 MHz, acetone-d_6) of gonioridleyilactam (**1**) 6

Figure S2 ^{13}C NMR (150 MHz, acetone-d_6) of gonioridleyilactam (**1**) 7

Figure S3 HSQC of gonioridleyilactam (**1**) 7

Figure S4 HMBC of gonioridleyilactam (**1**) 8

Figure S5 COSY of gonioridleyilactam (**1**) 8

Figure S6 NOESY of gonioridleyilactam (**1**) 9

Figure S7 LRESIMS (Low-resolution electrospray ionisation mass spectrometry) spectrum of gonioridleyilactam (**1**) 9

Figure S8 HRESIMS spectrum of gonioridleyilactam (**1**) 10

Figure S9 HRESIMS spectrum of Unit A (aristolactam II) in **1** 10

Figure S10 HRESIMS spectrum of Unit B (aristolactam I) in **1** 11

Figure S11 IR spectrum of gonioridleyilactam (**1**) 11

Figure S12 ^{1}H NMR (400 MHz, $CDCl_3$) of 3-hydroxymethyl-1-methyl-1*H*-benz[*f*]indole-4,9-dione (**2**) 12

Figure S13 ^{13}C NMR (100 MHz, $CDCl_3$) of 3-hydroxymethyl-1-methyl-1*H*-benz[*f*]indole-4,9-dione (**2**) 12

Figure S14 HSQC of 3-hydroxymethyl-1-methyl-1*H*-benz[*f*]indole-4,9-dione (**2**) 13

Figure S15 HMBC of 3-hydroxymethyl-1-methyl-1*H*-benz[*f*]indole-4,9-dione (**2**) 13

Figure S16 COSY of 3-hydroxymethyl-1-methyl-1*H*-benz[*f*]indole-4,9-dione (**2**) 14

Figure S17 NOESY of 3-hydroxymethyl-1-methyl-1*H*-benz[*f*]indole-4,9-dione (**2**) 14

Figure S18 HRESIMS spectrum of 3-hydroxymethyl-1-methyl-1*H*-benz[*f*]indole-4,9-dione (**2**) 15

Figure S19 IR spectrum of 3-hydroxymethyl-1-methyl-1*H*-benz[*f*]indole-4,9-dione (**2**) 15

Figure S20 The isolation and purification of isolated compounds **1-10** from the twig extract of *G. ridleyi.* 16

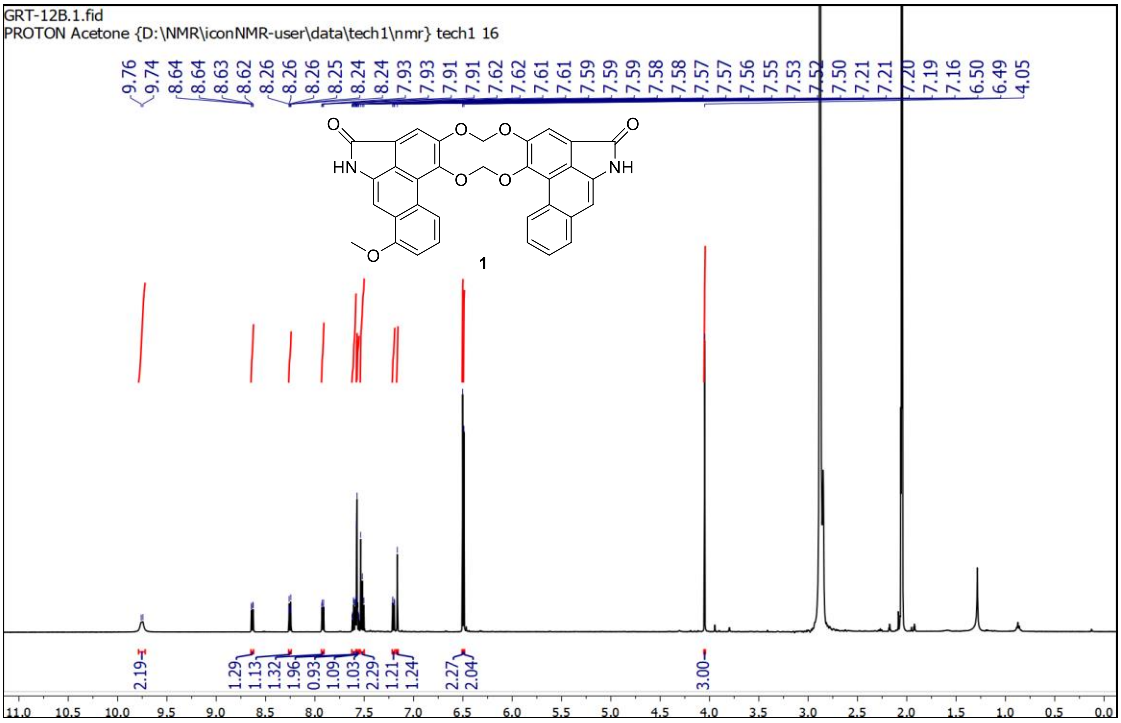

Figure S1 ^{1}H NMR (600 MHz, acetone-d_6) of gonioridleyilactam (**1**)

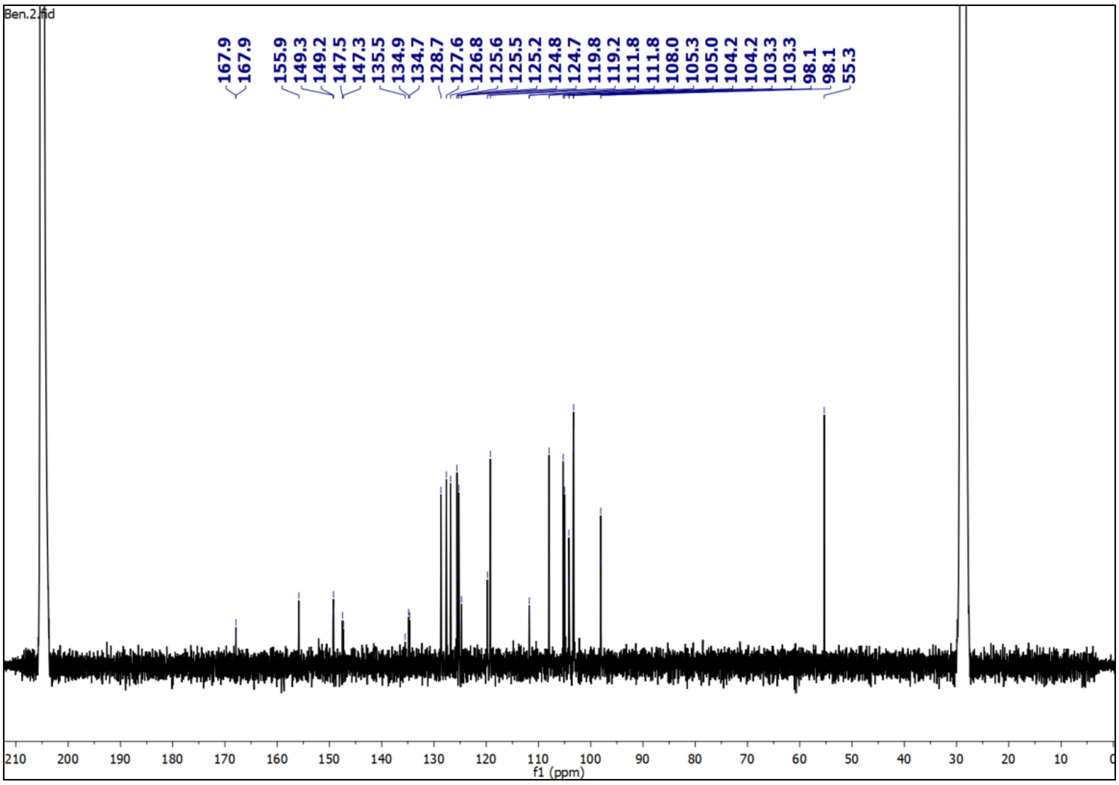

Figure S2 ^{13}C NMR (125 MHz, acetone-d_6) of gonioridleyilactam (**1**)

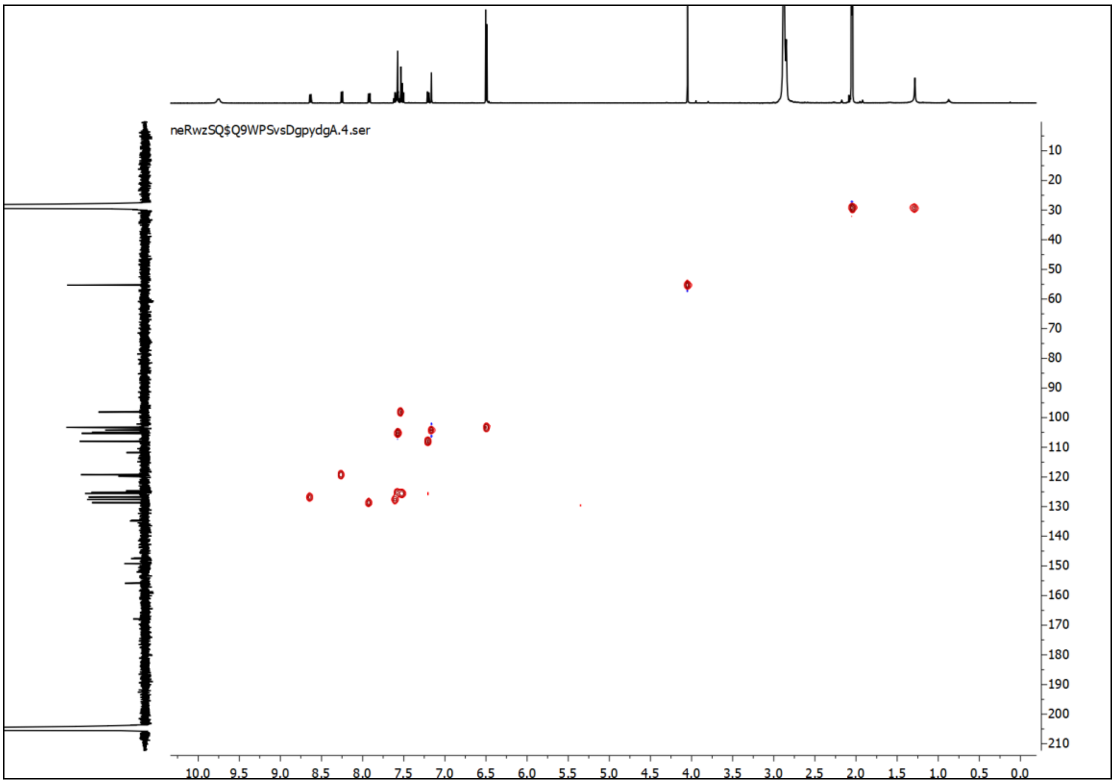

Figure S3 HSQC of gonioridleyilactam (**1**)

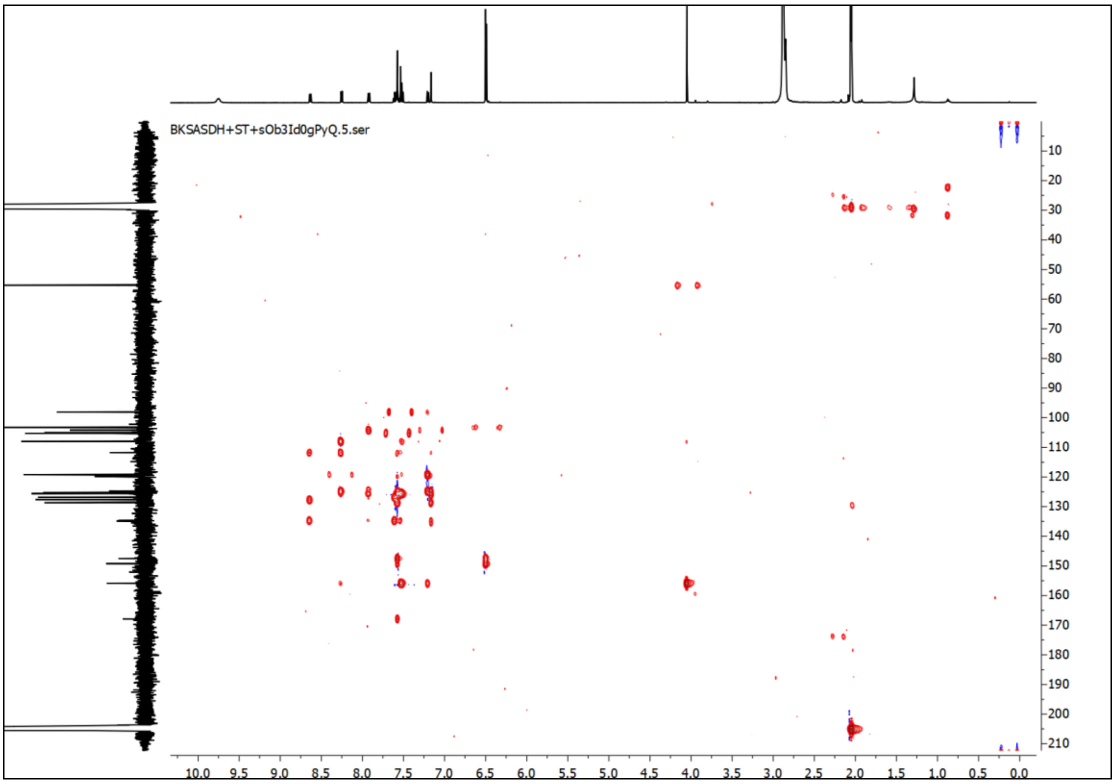

Figure S4 HMBC of gonioridleyilactam (**1**)

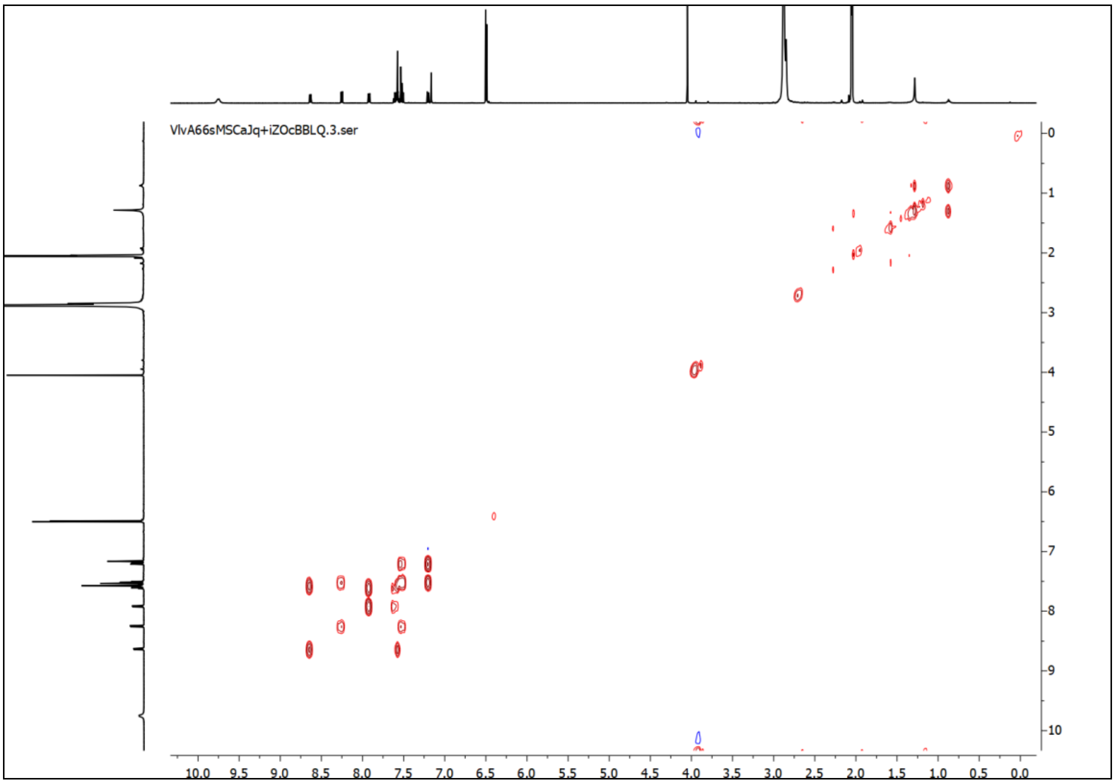

Figure S5 COSY of gonioridleyilactam (**1**)

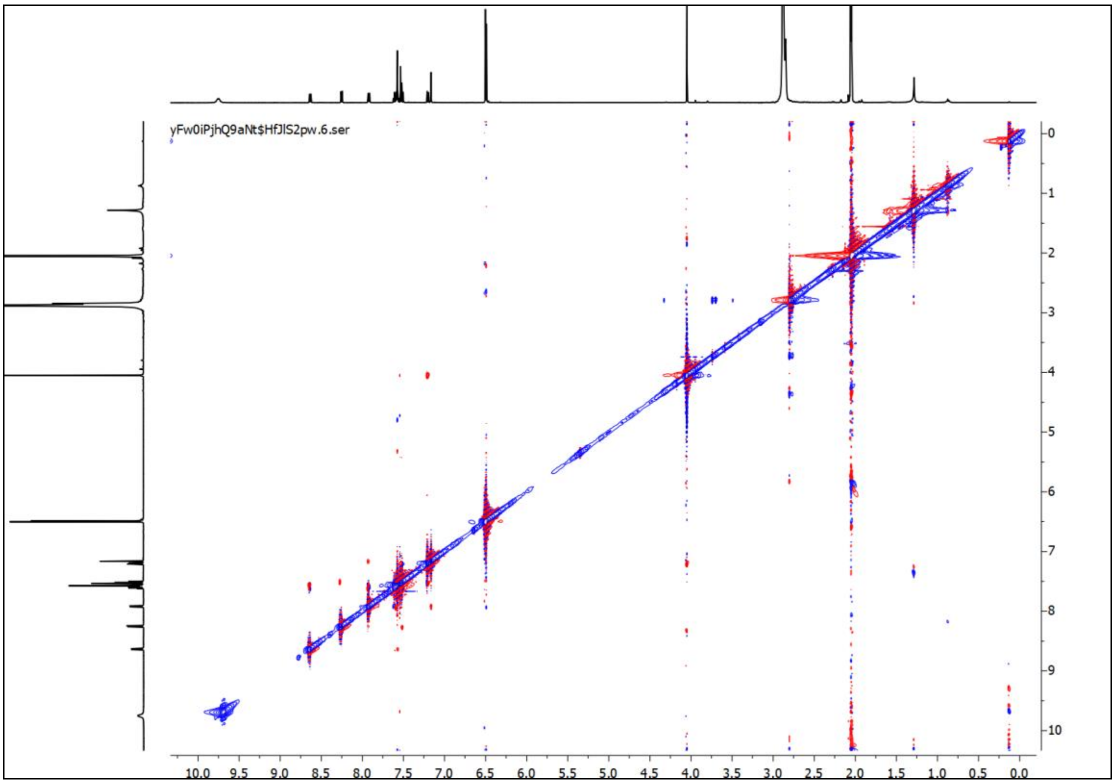

Figure S6 NOESY of gonioridleyilactam (**1**)

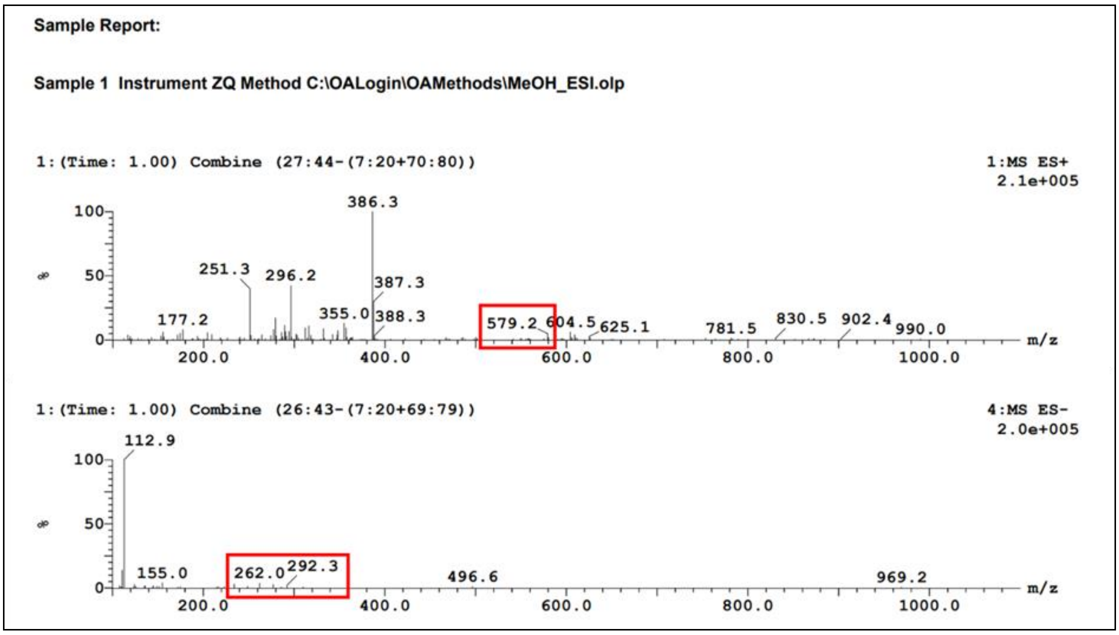

Figure S7 LRESIMS (Low-resolution electrospray ionisation mass spectrometry) spectrum of gonioridleyilactam (**1**)

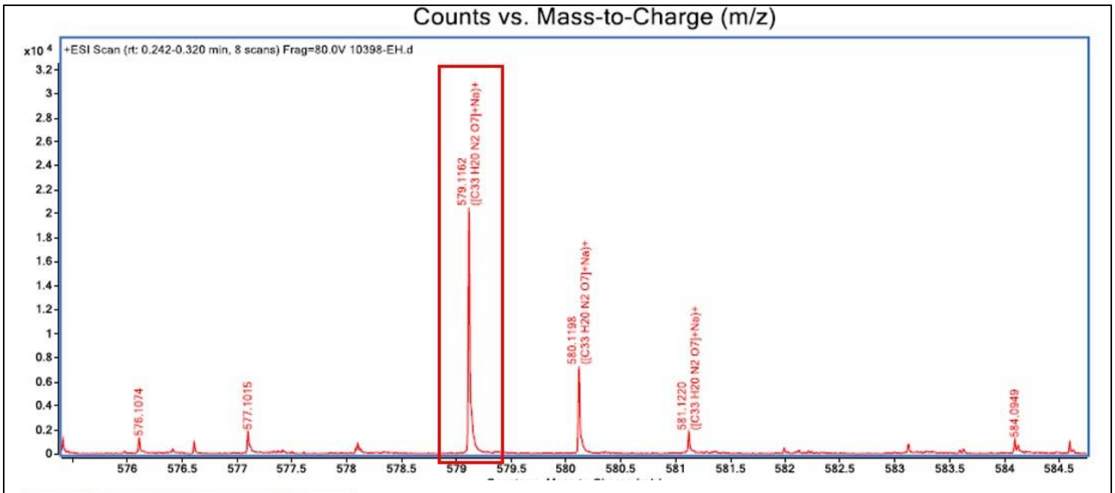

Formula Calculator Results

Formula	Mass	Tgt Mass	Diff (ppm)	Ion Species	Score
C33 H20 N2 O7	556.1271	556.1271	0.04	C33 H20 N2 Na O7	98.90
C32 H14 N9 O2	556.1272	556.1270	0.31	C32 H14 N9 Na O2	96.97
C31 H18 N5 O6	556.1271	556.1257	2.59	C31 H18 N5 Na O6	95.56
C34 H16 N6 O3	556.1271	556.1284	-2.23	C34 H16 N6 Na O3	94.02
C30 H22 N O10	556.1271	556.1244	4.85	C30 H22 N Na O10	88.41
C19 H22 N7 O13	556.1273	556.1276	-0.47	C19 H22 N7 Na O13	85.88

Figure S8 HRESIMS spectrum of gonioridleyilactam (**1**)

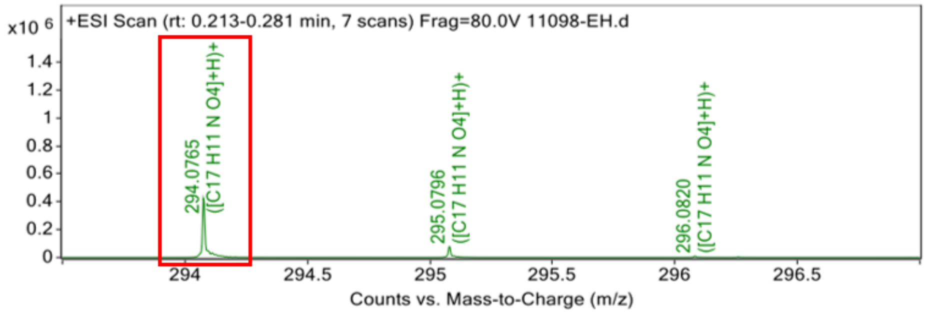

Formula Calculator Element Limits

Element	Min	Max
C	3	60
H	1	120
N	1	10
O	1	15

Formula Calculator Results

Formula	Mass	Tgt Mass	Diff (ppm)	Ion Species	Score
C17 H11 N O4	293.0692	293.0688	1.45	C17 H12 N O4	99.22
C16 H12 N3 O3	294.0877	294.0879	-0.44	C16 H13 N3 O3	47.56
C14 H10 N6 O2	294.0877	294.0865	4.12	C14 H11 N6 O2	42.65
C10 H18 N2 O8	294.1058	294.1063	-1.81	C10 H19 N2 O8	83.91
C9 H12 N9 O3	294.1059	294.1063	-1.30	C9 H13 N9 O3	83.77
C8 H16 N5 O7	294.1059	294.105	3.03	C8 H17 N5 O7	79.93

Figure S9 HRESIMS spectrum of aristolactam unit A of **1**

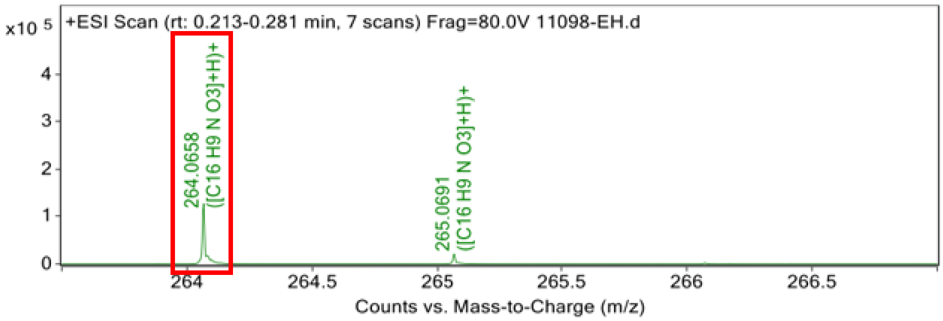

Formula Calculator Element Limits

Element	Min	Max
C	3	60
H	1	120
N	1	10
O	1	15

Formula Calculator Results

Formula	Mass	Tgt Mass	Diff (ppm)	Ion Species	Score
C16 H9 N O3	263.0585	263.0582	0.97	C16 H10 N O3	99.36
C6 H11 N6 O6	263.073	263.074	-3.66	C6 H12 N6 O6	44.10
C12 H13 N3 O4	263.0902	263.0906	-1.59	C12 H14 N3 O4	92.65
C10 H11 N6 O3	263.0903	263.0893	3.89	C10 H12 N6 O3	85.08

Figure S10 HRESIMS spectrum of aristolactam unit B of **1**

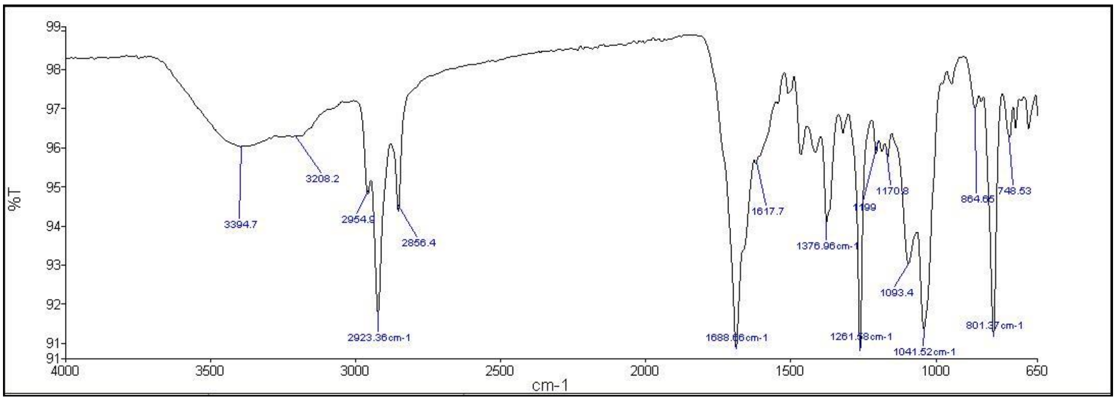

Figure S11 IR spectrum of gonioridleyilactam (**1**)

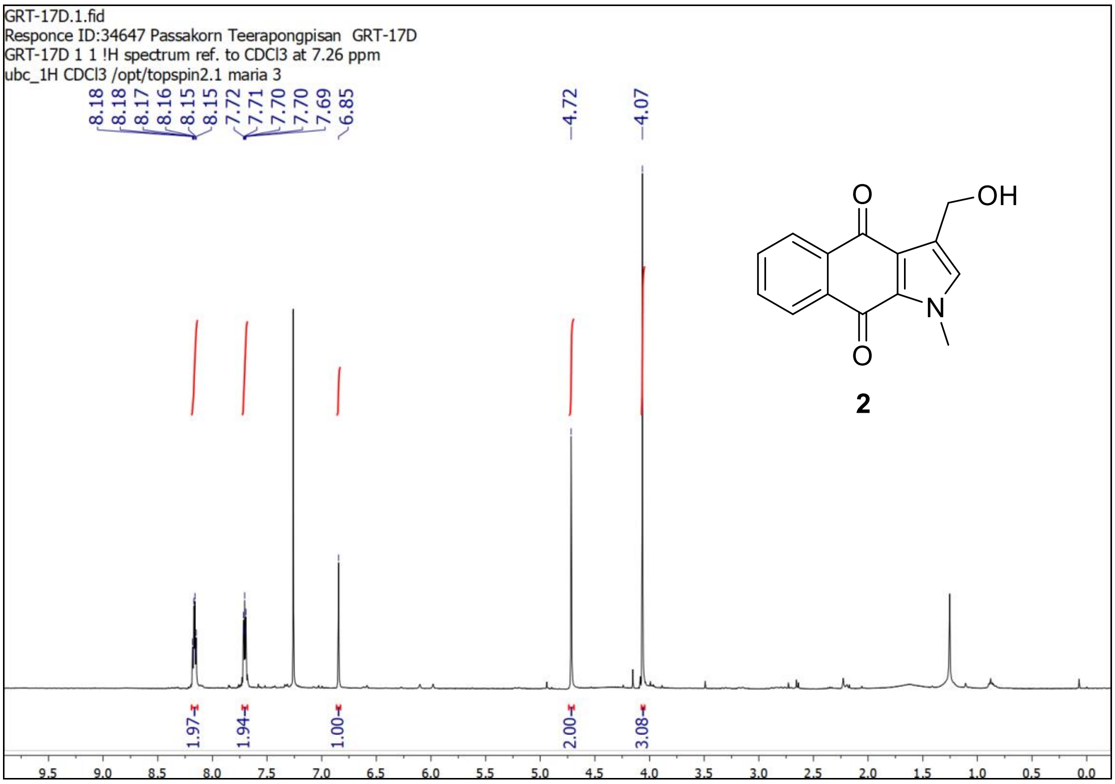

Figure S12 ^{1}H NMR (400 MHz, $CDCl_3$) of

3-hydroxymethyl-1-methyl-1*H*-benz[*f*]indole-4,9-dione (**2**)

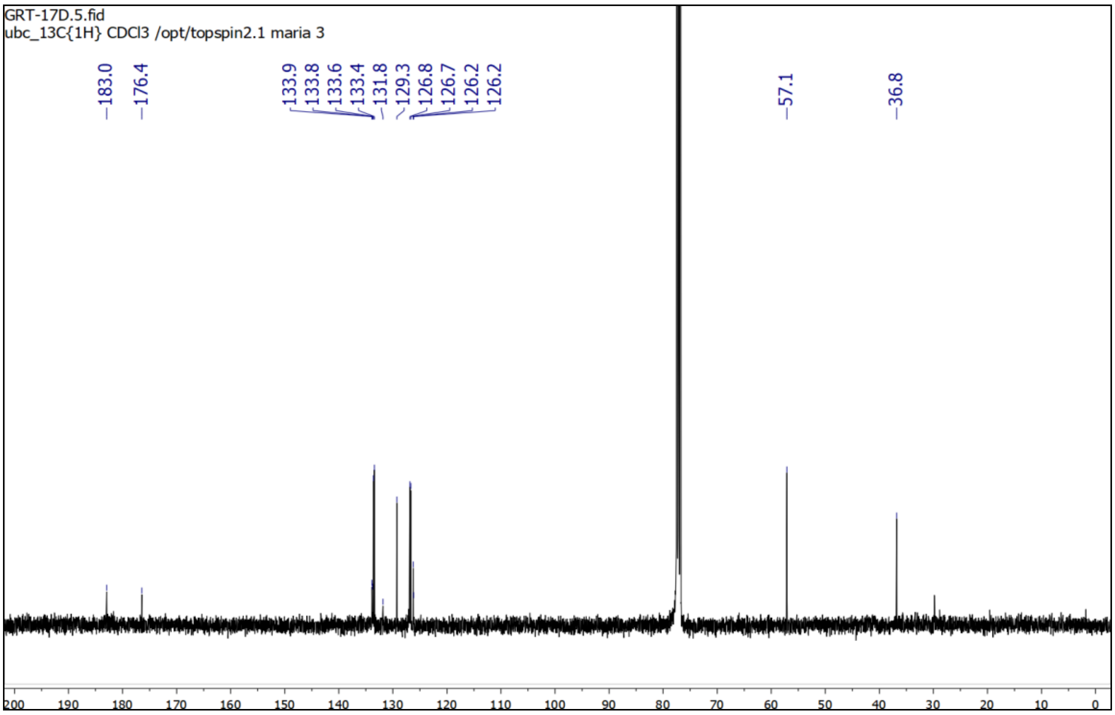

Figure S13 ^{13}C NMR (100 MHz, $CDCl_3$) of
3-hydroxymethyl-1-methyl-1*H*-benz[*f*]indole-4,9-dione (**2**)

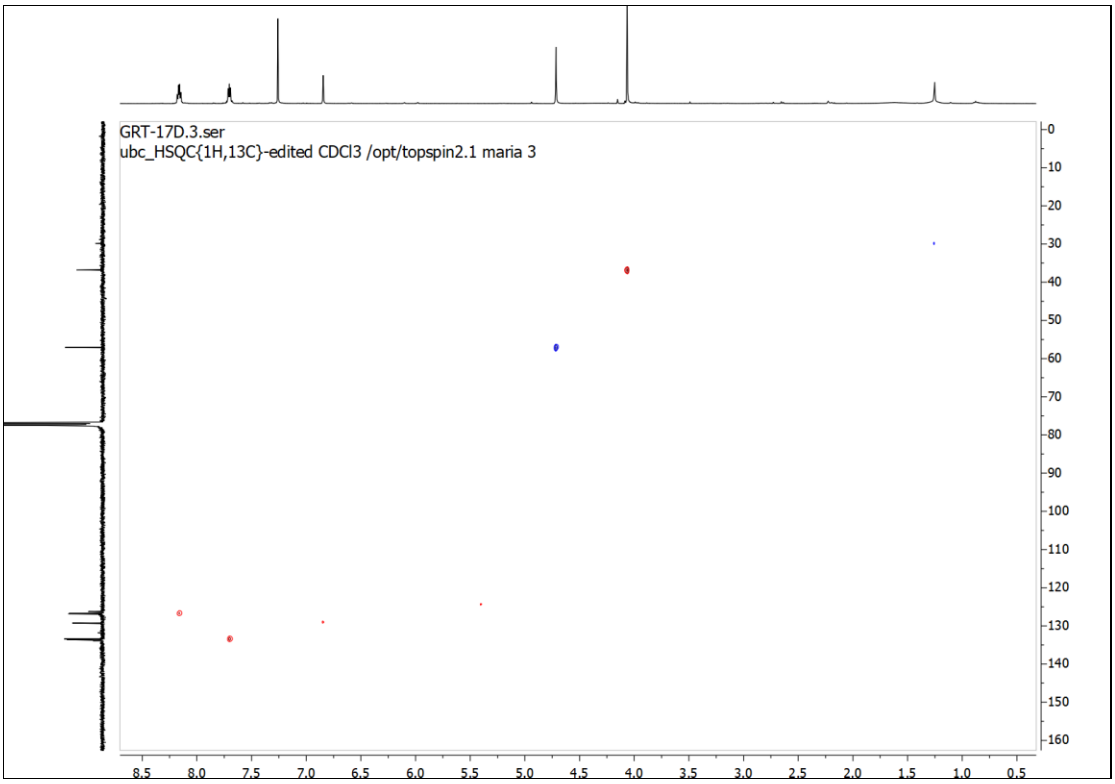

Figure S14 HSQC of 3-hydroxymethyl-1-methyl-1*H*-benz[*f*]indole-4,9-dione (**2**)

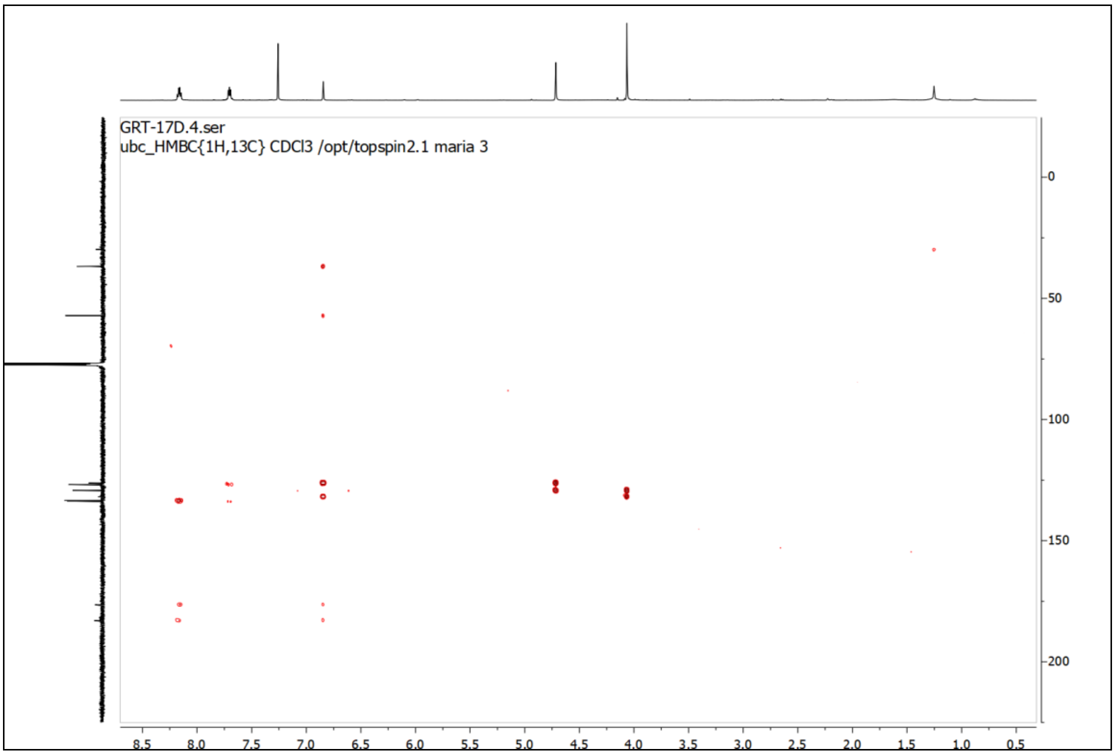

Figure S15 HMBC of 3-hydroxymethyl-1-methyl-1*H*-benz[*f*]indole-4,9-dione (**2**)

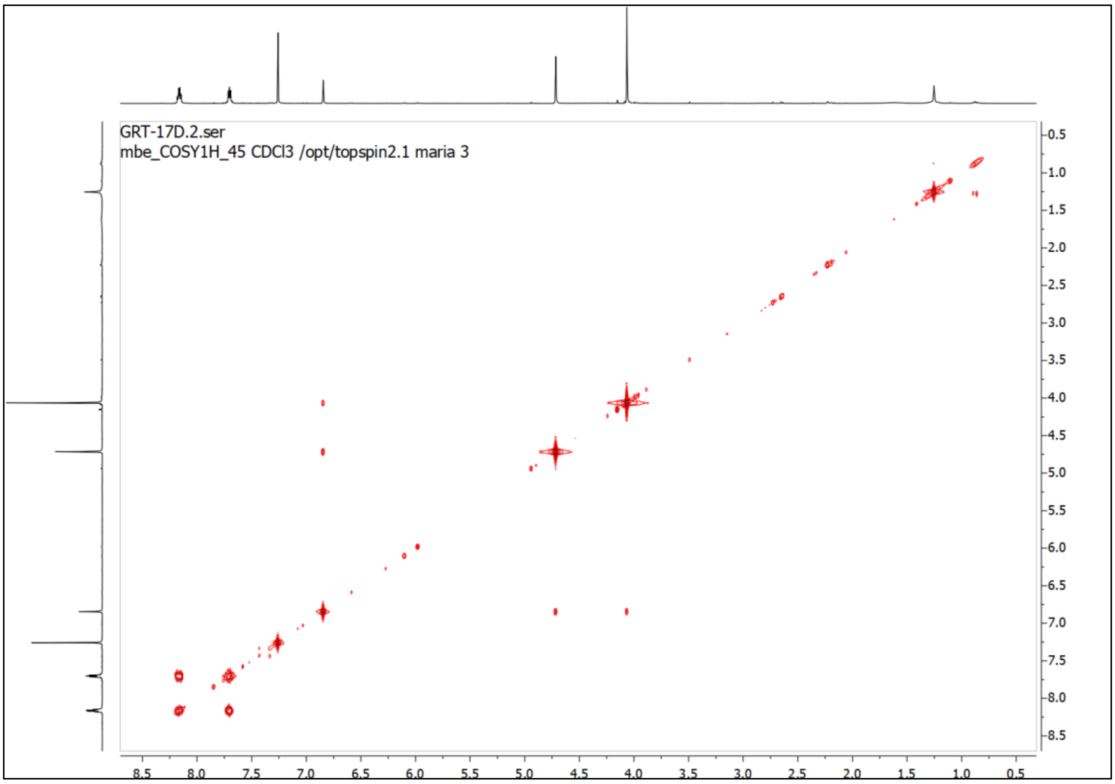

Figure S16 COSY of 3-hydroxymethyl-1-methyl-1*H*-benz[*f*]indole-4,9-dione (**2**)

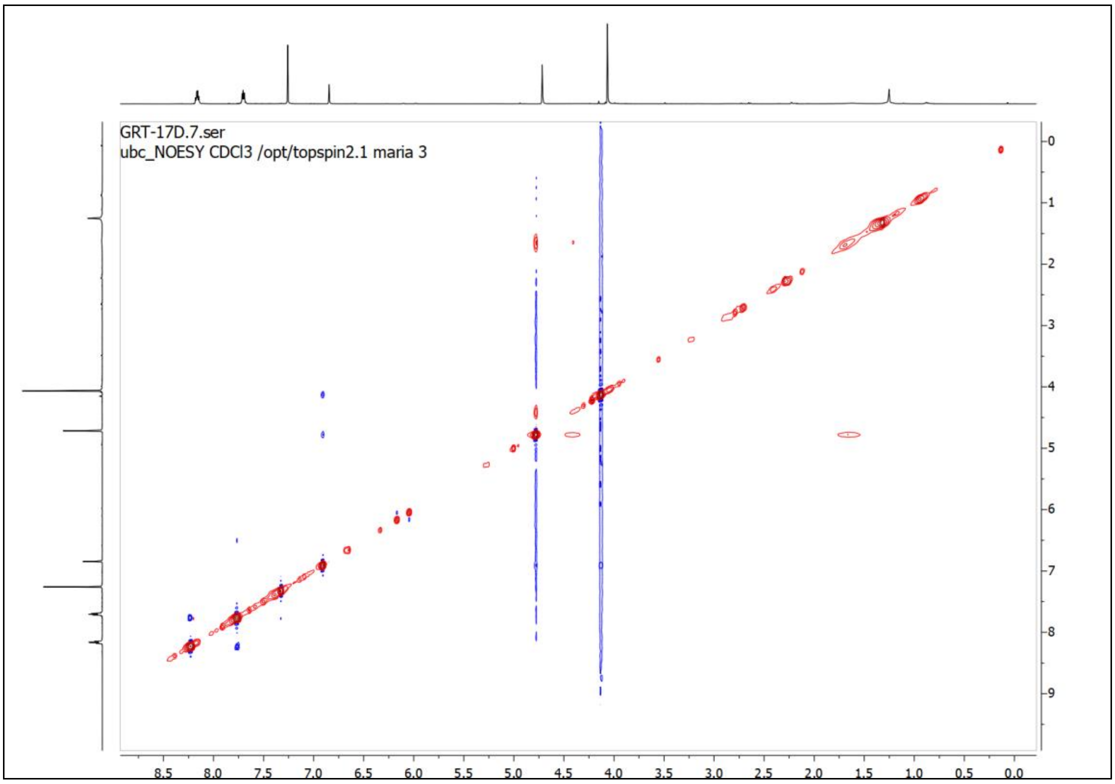

Figure S17 NOESY of 3-hydroxymethyl-1-methyl-1*H*-benz[*f*]indole-4,9-dione (**2**)

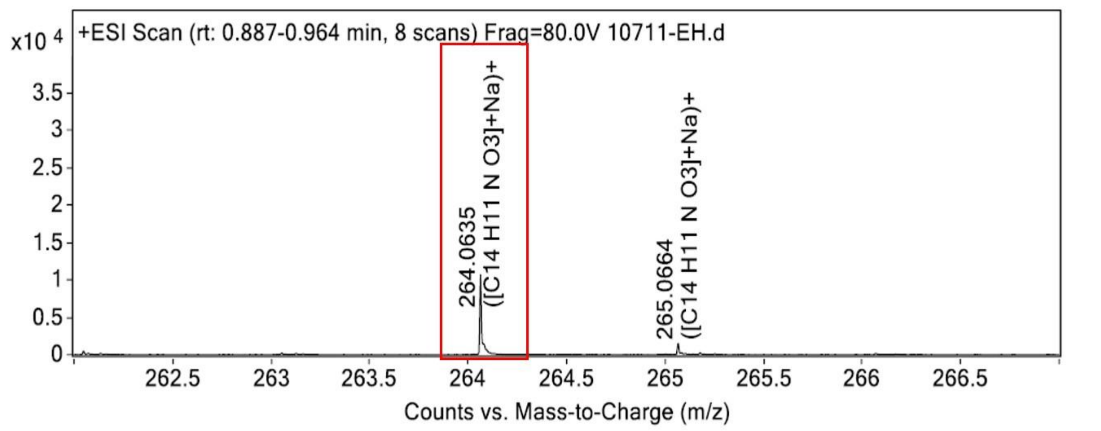

Formula Calculator Results

Formula	Mass	Tgt Mass	Diff (ppm)	Ion Species	Score
C14 H11 N O3	241.0742	241.0739	1.33	C14 H11 N Na O3	98.91

Figure S18 HRESIMS spectrum of 3-hydroxymethyl-1-methyl-1*H*-benz[*f*]indole-4,9-dione (**2**)

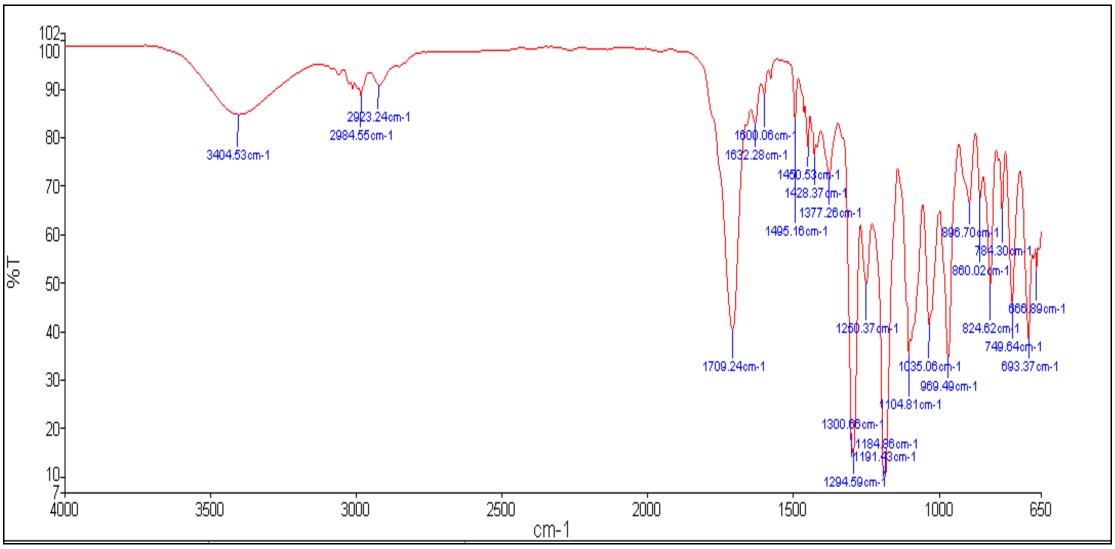

Figure S19 IR spectrum of 3-hydroxymethyl-1-methyl-1*H*-benz[*f*]indole-4,9-dione (**2**)

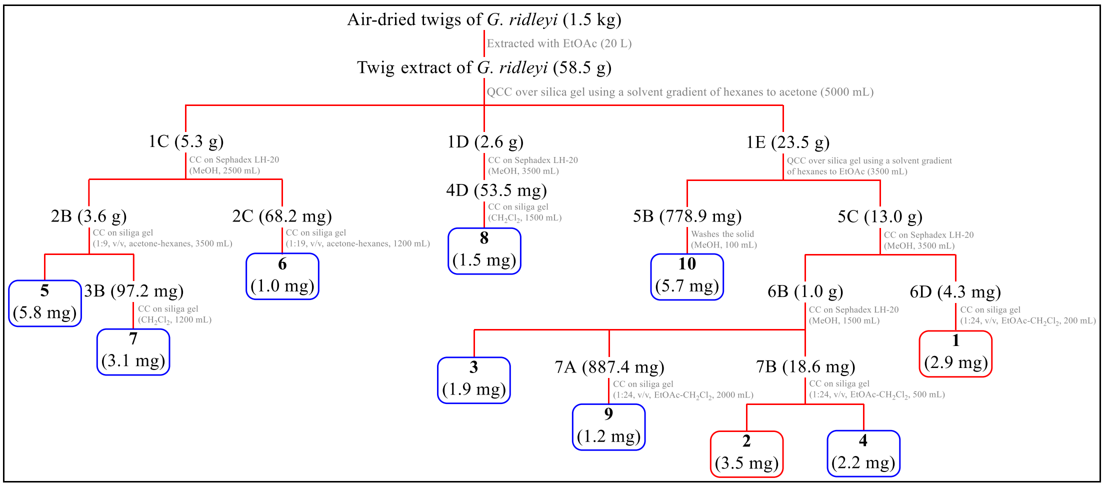

Figure S20 The isolation and purification of isolated compounds **1-10** from the twig extract of *G. ridleyi*
